# Protein kinase C epsilon deletion in AgRP neurons modulates hypothalamic glucose sensing and improves glucose tolerance in mice

**DOI:** 10.1016/j.molmet.2026.102320

**Published:** 2026-01-13

**Authors:** Amanda E. Brandon, Chenxu Yan, Xuan Zhang, Chi Kin Ip, Zhongmin Gao, Nicola J. Lee, Oana C. Marian, Alex Perez, Anthony S. Don, Herbert Herzog, Lewin Small, Yan-Chuan Shi, Carsten Schmitz-Peiffer

**Affiliations:** 1 Charles Perkins Centre, Faculty of Science, University of Sydney, NSW, Australia; 2 Neuroendocrinology Group, Garvan Institute of Medical Research, Sydney, NSW, Australia; 3 Neuroscience Division, Garvan Institute of Medical Research, Sydney, NSW, Australia; 4 Charles Perkins Centre, Faculty of Medicine and Health, University of Sydney, NSW, Australia; 5 St Vincent's Centre for Applied Medical Research, Sydney, NSW, Australia; 6 School of Clinical Medicine, Faculty of Medicine and Health, UNSW Sydney, Australia; 7 Garvan Institute of Medical Research, Sydney, NSW, Australia

**Keywords:** AgRP neuron, Glucose intolerance, High-fat diet, Hypothalamus, Protein kinase C epsilon

## Abstract

**Objectives:**

Global but not liver-specific deletion of protein kinase C epsilon (PKCε) improves glucose tolerance in fat-fed mice, suggesting that extra-hepatic tissues are involved. AgRP neurons within the arcuate nucleus (ARC) of the hypothalamus can affect glucose homeostasis acutely, in addition to their role in energy homeostasis. We therefore deleted PKCε specifically in AgRP neurons to examine its effects at this site.

**Methods:**

Fat-fed AgRP-PKCε^−/−^ mice were subjected to glucose tolerance tests and euglycaemic-hyperinsulinaemic clamps. c-Fos and tyrosine hydroxylase were used as markers to map neuronal activity in serial brain sections. Transcriptional changes in liver and adipose tissue were examined by qRT-PCR while alterations in protein levels and phosphorylation were determined by immunoblotting and mass spectrometry.

**Results:**

Fat-fed AgRP-PKCε^−/−^ mice exhibited improved glucose tolerance but not insulin sensitivity determined by clamp. c-Fos mapping demonstrated that glucose challenge resulted in greater activation of neurons in the paraventricular nucleus (PVN) in AgRP-PKCε^−/−^ mice, but reduced expression of tyrosine hydroxylase in the PVN, suggestive of reduced sympathetic outflow. This was associated with a reduction in hormone sensitive lipase phosphorylation and plasma fatty acid levels. Proteomic analysis indicated overlapping alterations in proteins and protein phosphorylation in adipose tissue and liver, consistent with changes in a common, potentially neuronal, cell type.

**Conclusions:**

Ablation of PKCε in AgRP neurons improves glucose homeostasis in fat-fed mice. This appears to be mediated through glucose sensing mechanisms, potentially reducing sympathetic outflow from the hypothalamus to tissues such as adipose, reducing lipolysis to indirectly lower hepatic glucose production.

## Highlights

• Deletion of protein kinase C epsilon (PKCε) in AgRP neurons improves glucose tolerance in fat-fed mice in an insulin-independent manner.
• PKCε deletion modulates circuits in the hypothalamus consistent with reduced sympathetic outflow via the paraventricular nucleus.
• Analysis of metabolites and protein phosphorylation supports changes in sympathetic input to adipose tissue, affecting hepatic glucose output.

## 1 Introduction

Type 2 diabetes has become a major global health problem, with 589 million adults affected globally, which is expected to reach 853 million by 2045 [1]. The disease is strongly associated with obesity and increased lipid availability, and is characterised by insulin resistance and pancreatic β-cell dysfunction, resulting in poor glycaemic control which in turn leads to debilitating complications [2].

In order to explain the development of type 2 diabetes, emphasis has previously been placed on peripheral mechanisms that can disrupt insulin release or insulin action. However, the significance of centrally-mediated events to the regulation of glucose metabolism, involving both insulin-dependent and independent mechanisms, has become apparent. Several glucose and insulin-sensitive regions of the brain have been identified, including the arcuate nucleus (ARC) of the hypothalamus which contains AgRP/NPY and POMC neurons [3,4]. The role of AgRP/NPY neurons, expressed exclusively in the ARC, in the control of food intake and energy homeostasis is well-established, and their contribution to the acute regulation of blood glucose levels is also emerging [[5], [6], [7]]. The activity of these neurons is modulated by insulin, which causes hyperpolarization and silencing, contributing to the suppression of hepatic glucose production (HGP) by the hormone [8,9]. Conversely, AgRP neurons are activated by low glucose levels in an AMPK-dependent manner [10], contributing to the counterregulatory response to hypoglycaemia. AgRP neurons are located in the ventromedial ARC, in close proximity to the median eminence, a circumventricular organ residing outside the BBB, making them ideally placed to respond directly to circulating hormones and nutrients, including glucose and lipids [11]. Signalling pathways modulated in AgRP neurons upon obesity and chronic lipid oversupply are therefore likely to impact on the control of blood glucose levels exerted by the brain. Further glucose-responsive regions involved in these effects have been identified in the hypothalamus, including the paraventricular nucleus (PVN), suprachiasmatic nucleus (SCN), dorsomedial nucleus (DMH) and especially the ventromedial nucleus (VMH), which appear to play key roles in enabling the hypothalamus to partition energy to meet peripheral needs [12,13].

The protein kinase C (PKC) family of lipid-activated kinases are strong candidates for mediating inhibitory effects of lipid accumulation on insulin action and glucose homeostasis [14]. PKC isoforms are classified into groups based on their structures and molecular regulation: the conventional PKCs have C1 and C2 domains that sensitize them to diacylglycerol (DAG) and Ca^2+^ respectively, whereas the novel PKCs have C1 domains but only C2-related regions incapable of binding Ca^2+^, rendering them DAG-sensitive but Ca^2+^-independent [15]. Meanwhile the atypical PKCs have only a partial C1 domain making them insensitive to both DAG and Ca^2+^ [15]. Because DAG levels are elevated in tissues such as liver and skeletal muscle of insulin resistant rodents and obese human subjects, it has long been proposed that activation of specific PKCs, especially the novel isoforms PKCδ, PKCε and PKCθ, leads to inhibitory crosstalk with the insulin signalling cascade, reducing glucose disposal or the suppression of hepatic glucose production [[16], [17], [18], [19], [20]]. Evidence from studies of mice deficient in specific novel PKC isoforms has supported causative roles for these kinases in the generation of insulin resistance. Whole-body PKCθ deletion improves IRS-1 tyrosine phosphorylation and insulin action in skeletal muscle of insulin resistant lipid-infused mice [21]. Global or liver-specific deletion of PKCδ improves glucose tolerance and insulin sensitivity and also reduces hepatic lipid accumulation in chow and fat-fed mice [22,23].

Ablation of PKCε in rodents also protects against the insulin resistance generated by a high fat diet. Thus fat-fed global PKCε knockout mice are almost as glucose tolerant as chow-fed animals, and do not exhibit diminished insulin sensitivity in the early stages of a high fat diet [24]. Consistent with this, rats treated with an antisense oligonucleotide against PKCε exhibit an improvement in hepatic insulin sensitivity when fed a high fat diet for 3 days [25]. Despite this agreement, direct inhibition of hepatic insulin signalling by PKCε is supported by the use of antisense oligonucleotides but not by genetic deletion [[24], [25], [26], [27], [28], [29]], and the importance of PKCε activity within the liver itself to whole body glucose metabolism is contested [30,31]. Recent use of tissue-specific PKCε knockout mice has indicated a role for the kinase in adipose tissue that partly explains the improvement in glucose homeostasis observed in global PKCε knockout mice, but also suggested that further sights of action remain to be identified [27].

PKCε gene expression is ubiquitous, and PKCε mRNA has been detected in AgRP-expressing neurons [32]. Given the involvement of AgRP/NPY neurons in several aspects of glucose homeostasis, we generated mice with an AgRP promoter-driven deletion of PKCε to examine the role of the kinase in these cells with respect to glycaemic control. We present evidence for the novel concept that PKCε at this central site contributes to the impairment of glucose homeostasis in high fat-fed mice, but that this is independent of insulin action and is associated with alterations in glucose sensing in the hypothalamus.

## 2 Materials and methods

### 2.1 Animals and high fat diet treatment

Ethical approval for mouse studies was granted by the Garvan Institute/St. Vincent's Hospital and the University of Sydney Animal Ethics Committees, which fulfill all the requirements of the NHMRC and the NSW State Government, Australia (approval numbers 16/20, 19/18, 19/22, 23/03 and 2023/2326). Animals were handled by trained personnel and all procedures carried out in accordance with the Australian code of practice for the care and use of animals for scientific purposes 8th Edition (2013). Mice were communally housed in a temperature-controlled room (22 ± 0.5 °C) with a 12 h light–dark cycle and fed a standard chow diet (10.88 kJ/g; 8% fat, 21% protein and 71% carbohydrate; Gordon's Specialty Stock Feeds, Yanderra, NSW, Australia).

Mice bearing *Prkce* floxed alleles (Flox-PKCε) were generated and maintained as heterozygous PKCε^fl/+^mice on a C57BL/6AusJ background as previously described [27]. To generate mice with a deletion of PKCε specifically in AgRP neurons of the ARC (AgRP-PKCε KO mice), we crossed PKCε^fl/+^ mice with transgenic C57BL/6 mice expressing *Cre* recombinase under the control of the *Agrp* gene promoter (AgRP-IRES-Cre mice), kindly provided by Joel Elmquist, University of Texas Southwestern Medical Center [33,34]. Genotyping was performed by PCR as described previously [27]. This tissue-specific PKCε-deficient line exhibited frequent heterozygous germline deletion of PKCε, detected through additional genotyping PCR reactions, as previously described for deletion of the kinase in adipose tissue [27]. Homozygous PKCε^fl/fl^, AgRP-IRES-Cre^+^ mice were relatively rare and were reserved for breeding. Experiments were therefore conducted on a heterozygous PKCε^fl/Δ^ background of both AgRP-EpsKO mice and littermate controls as previously [27]. Agrp reporter mice have been described previously [35].

At 10 weeks of age, male mice were fed a lard-based high-fat diet prepared in-house (19.67 kJ/g; 45% fat, 20% protein and 35% carbohydrate [16% sucrose]; based on Research Diets D12451, New Brunswick, NJ, USA) for 8 weeks [27]. Mouse body composition was measured after 7 weeks by NMR using a EchoMRI-900 Analyzer.

### 2.2 Glucose, insulin and pyruvate tolerance tests

For glucose tolerance tests, mice were fasted for 6 h (0800–1400), weighed and basal blood samples taken from the tail tip. EDTA (18 mM in NaCl 154 mmol/l) was added to blood samples (1/3 volume) which were kept on ice until plasma was obtained by centrifugation at 17,000 g for 2 min. Mice were injected i.p. with a 25% glucose solution (2 g/kg or 1 g/kg body weight after 1 or 8 weeks of high fat diet feeding respectively) and further blood samples taken at 15, 30 and 45 min. Blood glucose concentrations were measured using an Accu-Chek Performa glucometer (Roche) at 7.5, 15, 22.5, 30, 45, 60 and 90 min. Insulin was measured in mouse plasma by ELISA (CrystalChem). For insulin tolerance tests, mice fed a high fat diet for 8 weeks were fasted, weighed and basal blood glucose measured as above. Mice were injected i.p. with 0.5 U/kg insulin (Actrapid, Novo Nordisk) and blood glucose concentrations were measured at 20, 30, 45 and 60 min. For pyruvate tolerance tests, fat-fed mice were fasted and injected i.p. with 0.5 g/kg pyruvate and blood glucose concentrations were measured over 0–90 min. Free fatty acid (FFA) levels were measured in plasma collected at 0, 10 and 20 min using the NEFA C kit (Wako Diagnostics).

### 2.3 Metabolic phenotyping

Calorimetric data and physical activity were measured using indirect calorimetry TSE Systems PhenoMaster metabolic cages. Fat-fed mice were allowed one day for acclimatization before beginning 3 days of data collection. Oxygen consumption (VO_2_) and carbon dioxide production (VCO_2_) were measured to determine the respiratory exchange ratio (RER) and energy expenditure. Locomotor activity was converted from X and Y beam breaks to distance by the internal software. Due to the consistency of the high fat diet, food intake was measured manually by weighing remaining food, including smaller pieces on the cage floor, on two occasions for each mouse after metabolic cage housing. For fasting-refeeding studies, mice were weighed, fasted overnight and refed at 9 am, with weighing at 0, 0.5, 1, 2, 4, 8 and 24 h post refeeding.

### 2.4 Euglycaemic-hyperinsulinaemic clamps

Mice were fed high fat diets as above, with dual cannulation surgery performed after 7 weeks and hyperinsulinaemic-euglycaemic clamps performed 5–7 days later as previously described [27]. A [^3^H]glucose infusion allowed the rate of basal and clamp glucose disappearance (R_d_) to be determined using steady-state equations. Clamp hepatic glucose production was determined by subtracting the glucose infusion rate (GIR) from R_d_ [27,36].

### 2.5 Tissue harvest after glucose challenge

For investigation of interleukin 6 (IL-6) signalling in liver, mice were fed a high fat diet for 8 weeks, fasted overnight and challenged with glucose (1 g/kg ip). Livers were harvested 4 h later. Lysates for immunoblotting were prepared as previously [27], while RNA was isolated as described below. For investigation of gene expression in brown adipose tissue (BAT), mice were fed a high fat diet for 8 weeks, fasted as described for ipGTT above, and tissue harvested from unchallenged mice. For investigation of sympathetic stimulation and gene expression in white adipose tissue (WAT), mice were fed a high fat diet for 8 weeks, fasted and challenged with glucose (1 g/kg ip) as described for ipGTT above, and tissue harvested after 30 min. In each case, tissues were frozen under N_2_ (l) and stored at −70 °C until use.

### 2.6 Biochemical analyses of liver samples

Glycogen content was measured in livers obtained as described in 2.9. Liver powder (30 mg) was resuspended in 200 μl 1 M KOH and heated at 70 °C for 20 min. After addition of 75 μl saturated Na_2_SO_4_ and 1.725 ml 95% ethanol, glycogen precipitates were centrifuged at 17,000 g for 15 min at 4 °C. Pellets were resuspended in 200 μl MilliQ H_2_O and heated to 70 °C for 10 before addition of 1.8 ml 95% ethanol and centrifugation at 17,000 g for 15 min at 4 °C min. Pellets were resuspended in 250 μl 0.25 M acetate buffer, pH 4.75 containing 0.01 units amyloglucosidase (A1602, Sigma–Aldrich) at 37 °C for 16 h and glycogen digests then assayed as glucose equivalents using a glucose assay kit (GAGO20, Sigma–Aldrich).

Liver triglyceride (TG) content was assayed after extraction from 30 mg powdered tissue using the Folch method [37] and quantified as glycerol equivalents using an enzymatic colori-metric method (GPO-PAP reagent; Roche Diagnostics).

Liver cholesterol and cholesterol ester content were determined by LC-MS/MS after extraction of lipids from liver homogenates (∼100 μg protein) with the addition of 5 nmoles of d7-cholesterol (Avanti Research) and 5 nmoles of cholesterol ester (17:0) (Cayman Chemical) internal standards, using a two-phase methyl-tert-butyl ether (MTBE)/methanol/water protocol [38]. Dried lipid extracts were reconstituted in 360 μL of 25% 1-butanol:55% methanol:20% H2O containing 1 mM ammonium formate, 0.1% formic acid and 0.01% butylated hydroxytoluene. 300 μL was transferred to fused-insert glass HPLC vials. Lipids were detected in multiple reaction monitoring mode on a TSQ Altis triple quadrupole mass spectrometer coupled to a Vanquish HPLC (Thermo Scientific). Lipids were resolved on a Phenomenex Kinetex 3 × 100 mm (2.6 μm particle size) C8 column. Run time was 12.5 min, with a flow rate of 0.5 mL/min using the following binary gradient: 0–4.1 min 90% B; 4.1–4.2 100% B; 4.2–10.5 min 100% B; 10.5–10.6 min 90% B; 10.6–12.5 90% B, where mobile phase A was 1 mM ammonium formate and 0.1% formic acid in water, and mobile phase B was 1 mM ammonium formate and 0.1% formic acid in methanol. Cholesterol was detected as the [M + H–H2O]+ precursor *m/z* 369.4 and product ion *m/z* 161.1, and cholesterol esters were detected as the [M + NH4]+ precursor ion and product ion *m/z* 369.4. TraceFinder version 5.1 (ThermoFisher) was used for peak integration, and the molar amount of each lipid was calculated relative to its class-specific internal standard, then normalized to the protein content in each sample as determined by BCA assay.

### 2.7 Immunofluorescence

Mice were anaesthetised with an overdose of sodium pentobarbitone (120 mg/kg, i.p.) and brains fixed by cardiac perfusion with saline and 4% formaldehyde in PBS, as previously described [39,40]. Where indicated, mice were fasted as described for ipGTT above and challenged with glucose (1 g/kg) 30 min prior to perfusion. Brains were immediately removed and placed in 4% paraformaldehyde for 30 min and then in 30% sucrose solution in phosphate buffer overnight. Subsequently, brains were stored at −70 °C then sectioned coronally on a cryostat at 30 μm thickness. Sections were blocked in 5% normal goat serum (Merck #G9023) in PBS/0.2 % Triton X100 for 2 h at room temperature and incubated with primary antibodies overnight at 4 °C with gentle rocking. The antibodies used were mouse monoclonal PKCε antibody (1:50 dilution, Becton Dickinson 610086); rabbit polyclonal phospho-MARCKS (Ser152, Ser156) antibody (1:100 dilution, Thermo Fisher Scientific, #BS-3263 R); rabbit polyclonal cFos antibody (1:1200; Abcam ab190289) or sheep anti-mouse tyrosine hydroxylase antibody (Santa Cruz Biotechnology). All antibodies were diluted in PBS containing 0.1% Triton X-100, 5% normal goat serum and 0.1 % BSA. Sections were washed 3 times at RT for 10 min with PBS/0.2 % Triton X100 and then incubated for 2 h with appropriate secondary antibodies diluted 1:250 in antibody diluent: donkey anti-mouse Alexa Fluor 488 antibody, donkey anti-rabbit Alexa Fluor 488 antibody or donkey anti-sheep Alexa Fluor 488 antibody (Molecular Probes). Sections were washed 3 times as above and air-dried in the dark. Finally, sections were mounted on Superfrost slides (Menzel-Glaser, Braunschweig, Germany), cover slipped using Fluoroshield with DAPI (Merck, #F6057), and photographed using a Leica DM5500 fluorescent microscope (Leica, Germany). The quantification of phospho-MARCKS, cFos and Th signals was carried out using ImageJ software (version 2.16.0).

### 2.8 Quantitative real-time-PCR (qRT-PCR)

Livers were powdered under N_2_ (l). RNA was isolated from 50 mg of liver and 450–1150 ng RNA was reverse-transcribed to generate cDNA using the TaqMan Reverse Transcription Kit (Thermo Fisher) as per manufacturer's instructions. qRT-PCR was carried out using the Universal Probe Library (UPL) system on a LightCycler 480 Real-Time PCR system (Roche Applied Science, Germany). Quantification was calculated using the ΔΔCt method, with differences in cDNA input corrected by normalizing signals obtained with specific primers for the housekeeping gene cyclophilin B (Ppib).

**Primer Sequences (forward, reverse) and UPL Probe #:**

Ppib TTCTTCATAACCACAGTCAAGACC, ACCTTCCGTACCACATCCAT, #20.

IL-6 GCTACCAAACTGGATATAATCAGGA, CCAGGTAGCTATGGTACTCCAGAA #6.

Socs3, ATTTCGCTTCGGGACTAGC, AACTTGCTGTGGGTGACCAT #83.

G6pc TCTGTCCCGGATCTACCTTG, GAAAGTTTCAGCCACAGCAA #19.

Pck1 ATGTGTGGGCGATGACATT, AACCCGTTTTCTGGGTTGAT #105.

RNA was isolated from BAT and WAT using Trizol® Reagent (Sigma, St. Louise, MO, USA) following the manufacturer's protocol. One microgram (1 μg) of total RNA was reverse transcribed into cDNA using Superscript III First-Strand Synthesis System (Invitrogen, Mount Waverley, VIC, Australia). Quantitative real-time PCR using the forward and reverse primers listed below was carried out on a LightCycler Real-Time PCR system using Platinum Taq polymerase system (Thermo Fisher) following the manufacturer's instructions. The value obtained for each gene product was normalized by the ΔΔCT method to the housekeeping gene ribosomal protein L19 (Rpl19) expressed as a fold change of the value obtained for the controls.

**Primers (forward, reverse):**

Rpl19 CTCGTTGCCGGAAAAACA, TCATCCAGGTCACCTTCTCA.

Ucp1 GGCCTCTACGACTCAGTCCA, TAAGCCGGCTGAGATCTTGT.

Dio2 CTGCGCTGTGTCTGGAAC, GGAGCATCTTCACCCAGTTT.

Prdm16 CCTAAGGTGTGCCCAGCA, CACCTTCCGCTTTTCTACCC.

Cidea CTCCGAGTACTGGGCGATAC, ACCAGCCTTTGGTGCTAGG.

Adrb3 CAGCCAGCCCTGTTGAAG, CCTTCATAGCCATCAAACCTG.

Glut4 GGTGTGGTCAATACGGTCTTCAC, AGCAGAGCCACGGTCATCAAGA.

RNA was isolated from the hypothalamic block using TRIzol® reagent (Sigma) and an RNA Microprep kit (Zymo) according to the manufacturer's instructions. 300 ng of total RNA per sample was taken for cDNA synthesis using the iScript cDNA synthesis kit (Bio-Rad). qRT-PCR was carried out using the PowerUp SYBR Green master mix (Thermo Fisher) on the Lightcycler 480 System (Roche). The value obtained for each gene product was normalized by the ΔΔCT method to the geomean of three housekeeping genes Rpl19, beta (β)-actin (Actb), and hypoxanthine phosphoribosyltransferase 1 (Hprt1) expressed as a fold change of the value obtained for the controls.

**Primers (forward, reverse):**

Npy GAAAGCACAGAAAACGCCCCCAG, AAATGGGGCGGAGTCCAGCCTA.

Agrp AAGACAACTGCAGACCGAGC, GGACTCGTGCAGCCTTACAC.

Mc4r CCATCCTGATTGGAGTCTTT, TAAATGAGAGGGTCGATGAC.

Insr TCTTTCTTCAGGAAGCTACATCTG, TGTCCAAGGCATAAAAAGAATAGTT.

Lepr GTTCCAAACCCCAAGAATTG, TGCTCAAATGTTTCAGGCTTT.

Rpl19 CTCGTTGCCGGAAAAACA, TCATCCAGGTCACCTTCTCA.

Actb CTAAGGCCAACCGTGAAAAG, ACCAGAGGCATACAGGGACA.

Hprt1 ACAGGCCAGACTTTGTTGGA, ACTTGCGCTCATCTTAGGCT.

### 2.9 GT1-7 neuronal cell culture, adenovirus infection and treatment

GT1-7 mouse hypothalamic neuronal cells (Merck, mycoplasma negative) were cultured in Dulbecco's Modified Eagle Medium (DMEM) with 10% FBS, 500 IU/ml penicillin, 100 μg/ml streptomycin and 5.5 mM glucose, in a humidity-controlled 5% CO_2_ environment at 37 °C. They were seeded at 0.25 × 10^6^ cells per well in 12 well plates one day prior to infection with adenoviruses. Recombinant adenoviruses for the expression of kinase dead and constitutively active PKCε were prepared using the pAdEasy system as previously described [41] using well-characterised PKC cDNA constructs [42,43]. The amount of virus required to give 80–100% infection efficiency was individually determined by visualizing GFP expression. Two days following infection, cells were washed with PBS and incubated for 4 h in serum-free DMEM containing a range of glucose and 2-deoxyglucose (2DG) concentrations. Cells were then washed twice with ice-cold PBS, lysed in RIPA buffer supplemented with 125 mmol/l Tris–HCl, pH 6.8, 4% SDS, 20% glycerol, 10% dithiothreitol and 0.004% bromophenol blue, and heated at 70 °C for 10 min.

### 2.10 Immunoblotting

Whole cell extracts and tissue lysates were resolved by SDS-PAGE and immunoblotted as previously described [27]. Primary antibodies used were against total Myristoylated Alanine-Rich C-Kinase Substrate (MARCKS, Santa Cruz (N-19), sc-6454) and phospho-Ser159/163 MARCKS (Cell Signalling Technology #11992), total AMPKα (Cell Signalling Technology #2603) and phospho-Thr172 AMPKα Cell Signalling Technology # 2535), 14-3-3 protein (Santa Cruz (H-8), sc-1657) protein kinase C epsilon (Becton Dickinson, 610086), phospho-Tyr705 Signal transducer and activator of transcription 3 (Stat3, Cell Signalling Technology #9131), total STAT3 (Becton Dickinson, 610189), phospho-PKA substrate (Cell Signaling #9624), phospho-Ser133 CREB (Cell Signalling Technology #9198), phospho-Ser563 hormone sensitive lipase (HSL, Cell Signalling Technology #4139) uncoupling protein 1 (UCP1, Alpha Diagnostic International, UCP11-A) and vinculin (Abcam, ab73412). Horseradish peroxidase-linked secondary antibodies used were donkey anti-rabbit (Jackson, 711-035-152), sheep anti-mouse (Biostrategy, GEHEN-A931) or donkey anti-goat (Santa Cruz, sc-2020) as appropriate. Chemiluminescence, using Western Lightning Plus chemiluminescent substrate (Perkin–Elmer), was measured using a Bio-Rad ChemiDoc Touch Imaging System, and analysed by densitometry with ImageJ v2.3.0/1.53f.

### 2.11 Proteomic and phosphoproteomic analysis

Mice that had been fed a high fat diet for 8–22 weeks were challenged with glucose as detailed for glucose tolerance tests above, and tissues harvested 30 min later. Liver and adipose tissue proteins were extracted and 1 μg samples prepared for total proteomic and phosphoproteomic analysis according to the EasyPhos protocol [44], as described previously [45]. Raw data and the output of Spectronaut and DIANN searches were uploaded to the ProteomeXchange Consortium via the PRIDE [46] partner repository with the dataset identifier PXD065137:

Reviewer account details:

Username: reviewer_pxd065137@ebi.ac.uk.

Password: EzZ8A665MlEK.

All proteomic data were analysed in R as described [45], using limma [47] to test for differential abundance. Regarding the comparison of immunoblot and phosphoproteomic data for HSL, although post LC-MS/MS analysis identified Ser559 rather than Ser557 (mouse sequence, recognised by the phospho- Ser563 HSL antibody above), as the residue affected by PKCε deletion, this is likely due to the complexity of assigning phosphorylation sites in the tryptic peptide, given the three serine residues present. Phosphorylation site motif analysis was carried out using PhosphoSitePlus v 6.8.0 [48]. Analysis of the enrichment of Gene Ontology Biological Process terms for phosphosites or proteins with differing abundance in AgRP-PKCε KO and littermate control mice (nominal p values < 0.05) was performed using ToppCluster [49] and only enrichment terms with FDR <0.05 were considered (adjusted for multiple comparisons using Benjamini-Hochberg correction). Significantly enriched terms were manually selected and curated to avoid repetition of similar terms, and the resulting networks manually arranged using Cytoscape [50].

### 2.12 Statistical analysis

All data are expressed as means ± SEM. Differences between groups were assessed by Student's t-test, one-way or two-way ANOVA with Tukey post hoc test or Šídák's multiple comparisons test respectively, using GraphPad Prism v9.3.1. Statistical significance was defined as P < 0.05.

## 3 Results

### 3.1 PKCε deletion in AgRP neurons protects against diet-induced glucose intolerance but does not affect insulin sensitivity

**Figure 1:**
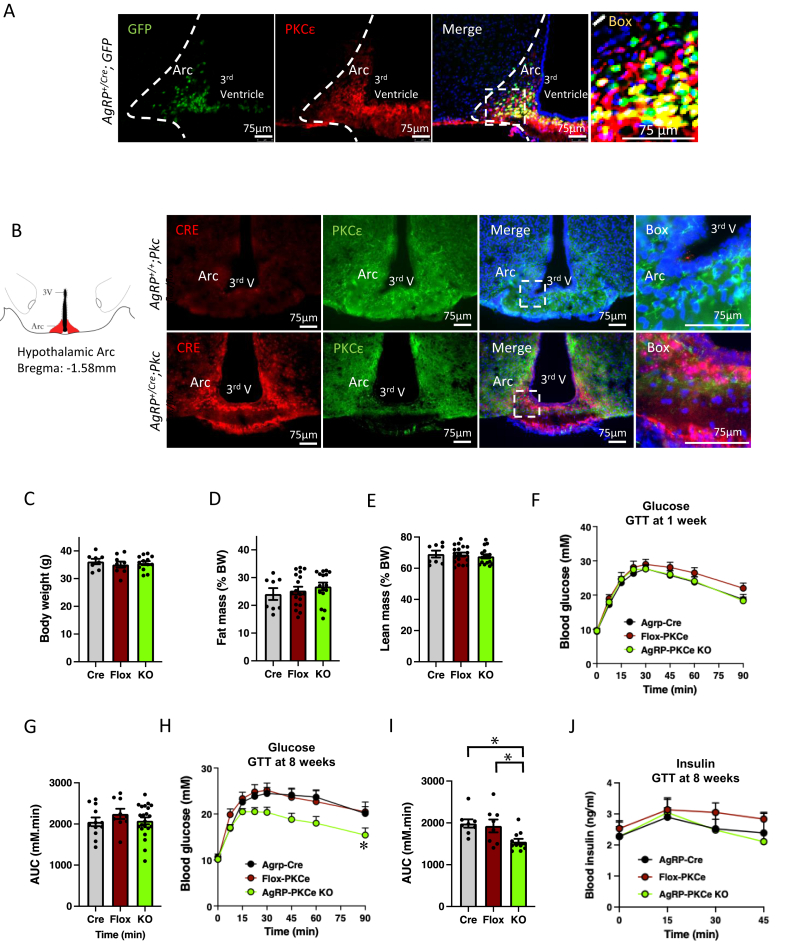
Deletion of PKCe in AgRP neurons improves glucose tolerance of fat-fed mice but does not affect body weight or composition. (**a**) Micrographs of coronal sections from the Arc. GFP expression in the NPY-GFP reporter mice is green, immunostaining for PKCe is red, and nuclear staining with DAPI is blue. The third panel shows a merged image and the fourth panel is a higher magnification of the boxed area; GFP-and PKCe-positive cells are shown in yellow. Images shown are typical of three biological replicates. (**b**) Representative photomicrographs showing expression of Cre recombinase (red) in the ARC of AgRP-PKCe KO mice (lower panels) and reduced expression of PKCe (green) in these mice compared to Flox-PKCe mice (upper panels); cell nuclei were stained with DAPI (blue). The box outlined in the third panels was enlarged (fourth panels) to highlight depletion of PKCe. 3V = 3^rd^ Ventricle; ARC = Arcuate Nucleus. Total body weight (**c**), fat mass (**d**) and lean mass (**e**) measured at 7 weeks of diet feeding of AgRP-Cre (Cre, n=8), Flox-PKCe (Flox, n=9) and AgRP-PKCe KO (KO, n=13) mice. Glucose tolerance was measured after 1 week (**f**) and 8 weeks (**h**) of high fat diet feeding; ANOVA: ∗P<0.05, effect of genotype. (**g**) Area under the glucose curve from the 1 week ipGTT shown in (**f**). (**i**) Area under the glucose curve from the 8 week ipGTT shown in (**h**) (ANOVA: ∗P<0.05 versus AgRP-Cre or Flox-PKCe). (**j**) plasma insulin levels during the ipGTT shown in (**e**).

To investigate the expression and distribution of PKCε protein in the arcuate nucleus (ARC), with a particular focus on its potential co-localization with AgRP neurons, we immunostained coronal sections of the ARC from Agrp reporter mice (*Agrp*^*+/Cre*^;TRAP^lox/lox^) [35]. These mice express EGFP fused to ribosomal protein L10a specifically in AgRP neurons. Along the coronal axis of the median eminence within the ARC, there was strong expression of PKCε protein localized proximally to the 3rd ventricle (Fig. 1A). Interestingly, approximately 90 percent of the AgRP-expressing neurons co-express PKCε (Fig. 1A, yellow label), but not all PKCε-positive cells express AgRP. To specifically investigate whether PKCε signalling in AgRP neurons plays a significant role in regulating systemic glucose homeostasis, we next crossed mice with a “floxed” PKCε gene (*Pkce*^*flox/flox*^) [27]with transgenic mice expressing *Cre* recombinase under control of the AgRP gene promoter (*Agrp*^*+/Cre*^) to generate AgRP neuron-specific PKCε knockout (*Agrp*^*+/Cre*^, *Pkce*^*flox/flox*^; AgRP-PKCε KO) mice. The specificity of Cre expression in AgRP neurons in AgRP-Cre mice has been confirmed previously [33,35]. Importantly, we demonstrated that deletion of the kinase at the protein level is specifically observed only in the *Agrp*-mediated Cre positive neurons, evident by the complete loss of PKCε signal in the cytoplasmic space (Fig. 1B, lower panels), confirming the successful generation of AgRP neuron-specific PKCε knockout mice.

To determine metabolic effects driven by the PKCε deletion in AgRP neurons, AgRP-PKCε KO and littermate control mice were fed a high-fat diet for 8 weeks. Deletion of the kinase resulted in reduced expression of AgRP, but not of NPY nor the MC4, leptin and insulin receptors, in the hypothalamus (Supplementary Fig. 1), consistent with cell-specific deletion. However, altered AgRP expression did not lead to changes in food intake or fasting-refeeding behaviour (Supplementary Fig. 2a,b), nor in RER, energy expenditure or locomotor activity (Supplementary Fig. 2c-h). These similar metabolic parameters are in agreement with the comparable body weight and composition of the mice (Fig. 1C–E). We next assessed glucose tolerance, using both Flox-PKCε and also AgRP-Cre control mice to enable assessment of the possible contributions of the floxed allele and the introduced Cre transgene. ipGTTs performed after 1 week of fat feeding did not highlight any differences in glucose tolerance (Fig. 1F,G). However, after 8 weeks on a fat diet, AgRP-PKCε KO mice exhibited improved glucose tolerance compared to Flox-PKCε and AgRP-Cre control mice (Fig. 1H,I), suggesting that PKCε signalling within AgRP neurons may be a driver for the impairment in glucose tolerance upon long-term positive energy balance. This was not explained by any differences in insulin excursions during the ipGTT (Fig. 1J).

**Figure 2:**
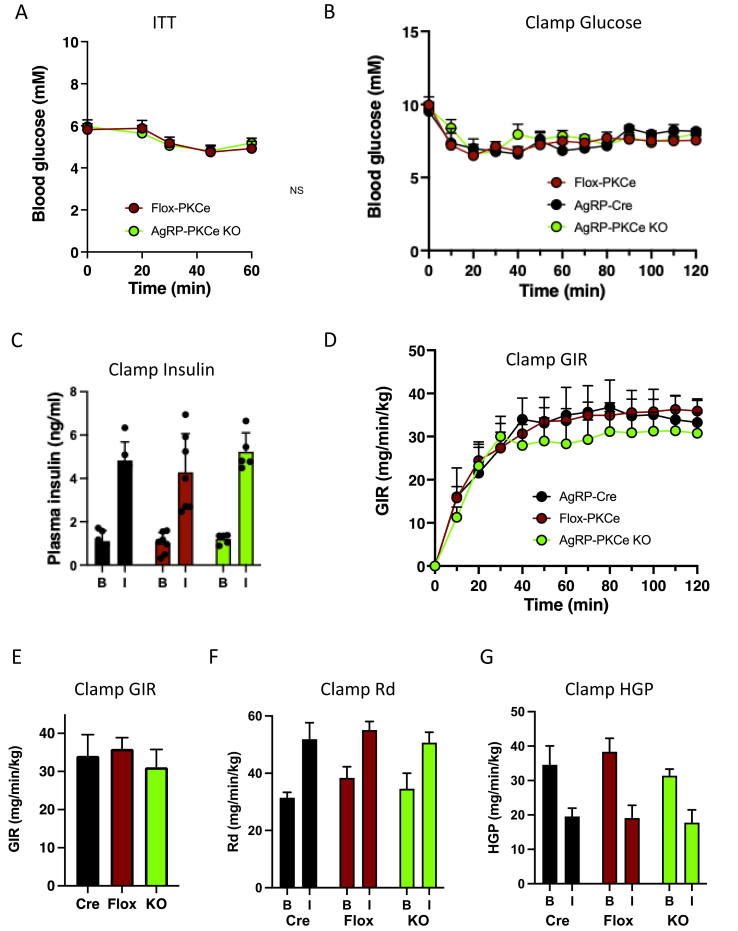
Deletion of PKCe in AgRP neurons does not affect insulin sensitivity in fat-fed mice. (**a**) Insulin tolerance test (ITT) in Flox-PKCe (n=14) and AgRP-PKCe KO mice (n=9) after 8 weeks of high fat diet feeding. (**b**) Blood glucose, (**c**) plasma insulin and (**d**) glucose infusion rates (GIR) during euglycaemic-hyperinsulinaemic clamps (Clamp) in AgRP-Cre (n=5), Flox-PKCe (n=8) and AgRP-PKCe KO mice (n=6) after 8 weeks of high fat diet feeding. (**e**) Mean GIR during the steady state phase of the clamps. (**f**) Whole body glucose disappearance (Rd) and (**g**) hepatic glucose output (HGO) during basal (B) and insulin-stimulated (I) conditions. NS = not significant.

We then determined whether the improved glucose tolerance observed in chronically fat-fed AgRP-PKCε KO mice was due to an enhancement of insulin sensitivity. Insulin tolerance tests performed after 8 weeks of diet feeding indicated that there were no gross changes in the response to a bolus of insulin (Fig. 2A). To measure insulin sensitivity in more detail, we subjected mice to euglycaemic-hyperinsulinaemic clamps (Fig. 2B–G). The glucose infusion rates required to maintain euglycaemia upon hyperinsulinaemia were not different between AgRP-PKCε KO mice and littermate controls (Fig. 2D,E), and there was also no difference in glucose disposal (R_d_) or hepatic glucose production (HGP) (Fig. 2F,G). Together with the unchanged insulin levels during the ipGTT (Fig. 1J), these results indicate that the improved glucose tolerance observed in AgRP-PKCε KO mice occurs in an insulin-independent manner and is likely to be mediated through a neuronal relay mechanism.

### 3.2 Effect of PKCε on AMPK and MARCKS phosphorylation in hypothalamic neurons

**Figure 3:**
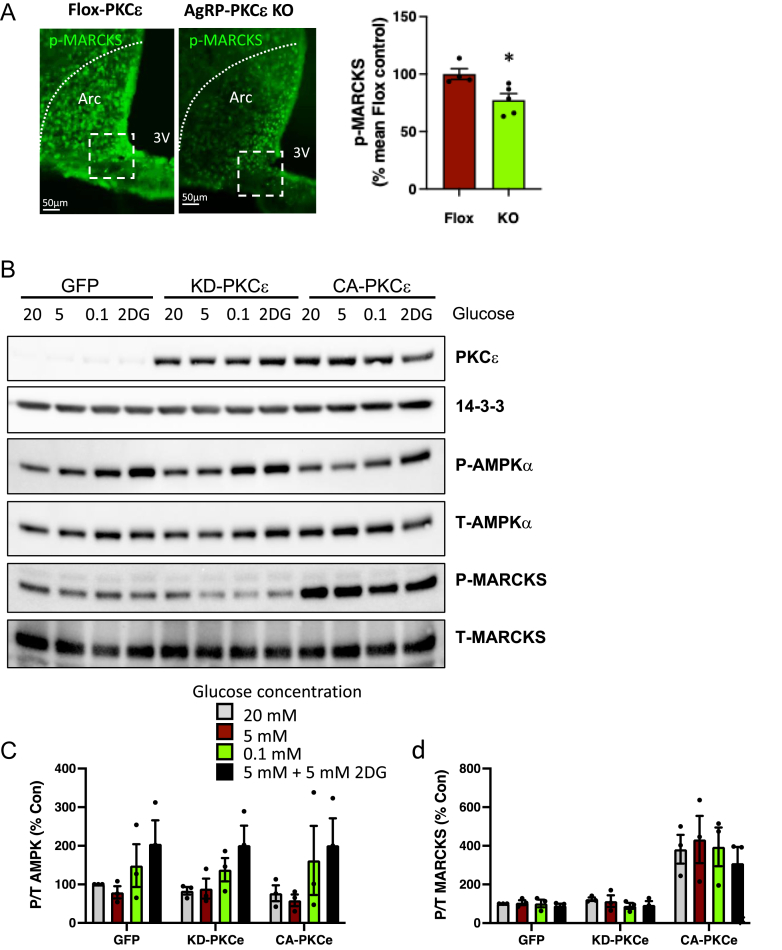
Effect of PKCe on MARCKS phosphorylation in the ARC in glucose-challenged mice and in GT1-7 cells exposed to glucose. (**a**) AgRP-PKCe KO mice and Flox-PKCe controls were challenged with glucose (1g/kg ip) and brains fixed by cardiac perfusion with saline/formaldehyde after 30 min. Cryosections were subjected to immunofluorescence using antibodies against phospho-MARCKS. 3V, 3^rd^ ventricle. Sections shown are typical of 4 biological replicates per group. Intensity of phospho-MARCKS staining was quantified within the defined areas of interest that corresponded to the area of Cre expression and PKCe depeletion in Fig. 1b; Student’s t-test, ∗P< 0.05. (**b**) GT1-7 cells were infected with adenovirus mediating the co-expression of GFP and kinase-dead (KD) or constitutively active (CA) PKCe as indicated. Cells were exposed to the concentrations of glucose indicated, or with 5 mM glucose as well as 5 mM 2-deoxyglucose (2DG), for 4 hours. Cell extracts were subjected to SDS-PAGE and immunoblotting with the antibodies indicated. Representative immunoblots of three experiments carried out in triplicate are shown. (**c**) The results of densitometry (means ± SEM) of AMPKa phosphorylation. Two-way ANOVA: P<0.01, main effect of glucose. (**d**) The results of densitometry (means ± SEM) of MARCKS phosphorylation. Two-way ANOVA: P<0.001, main effect of PKCe. One-way ANOVA: ∗ P<0.05, ∗∗ P<0.01 vs GFP.

We next investigated how the improved glucose tolerance we observed in fat-fed AgRP-PKCε KO mice might be mediated by circuits linked to AgRP neurons. Firstly, to investigate PKCε signalling within AgRP neurons, we examined the level of phosphorylation of the PKC substrate myristoylated alanine-rich C kinase substrate (MARCKS). MARCKS phosphorylation is a marker for PKC activity including PKCε [51], and we initially used it here as a functional readout of altered PKCε expression. We examined MARCKS phosphorylation by immunostaining the ARC of AgRP-PKCε KO mice and Flox-PKCε littermate controls after glucose challenge, which indicated that deletion of PKCε in AgRP neurons reduced MARCKS phosphorylation when compared to that observed in controls (Fig. 3A).

We subsequently examined signalling in neurons that could link PKCε with impaired glucose homeostasis. A reduction in glucose availability affects the polarization of AgRP neurons through the activity of AMP-dependent kinase (AMPK), leading to increased HGP [10,52]. PKCε has been implicated in the upregulation of AMPK activity [53], leading to the possibility that PKCε activation in neurons of fat-fed mice promotes AMPK-mediated effects at higher glucose concentrations, inappropriately increasing glucose production. We tested the effects of kinase dead or constitutively active PKCε overexpression on AMPK phosphorylation in hypothalamic GT1-7 cells, an *in vitro* model for AgRP neurons [54], exposing the cells to a range of glucose concentrations or to 2-deoxyglucose. A reduction in glucose availability caused an increase in AMPK phosphorylation as expected (Fig. 3B,C). However, this was not altered by the overexpression of either kinase dead or constitutively active mutants of PKCε. In contrast, constitutively active but not kinase dead PKCε strongly increased the phosphorylation of the PKC substrate MARCKS, but this was not sensitive to the prevailing glucose concentration (Fig. 3C,D). This pattern of MARCKS phosphorylation not only validates the overexpression of active PKCε in this model and its lack of effect on AMPK, but is also consistent with a role for reduced MARCKS phosphorylation in the effects of PKCε deletion on glucose homeostasis, independently of prevailing glucose levels.

**Figure 4:**
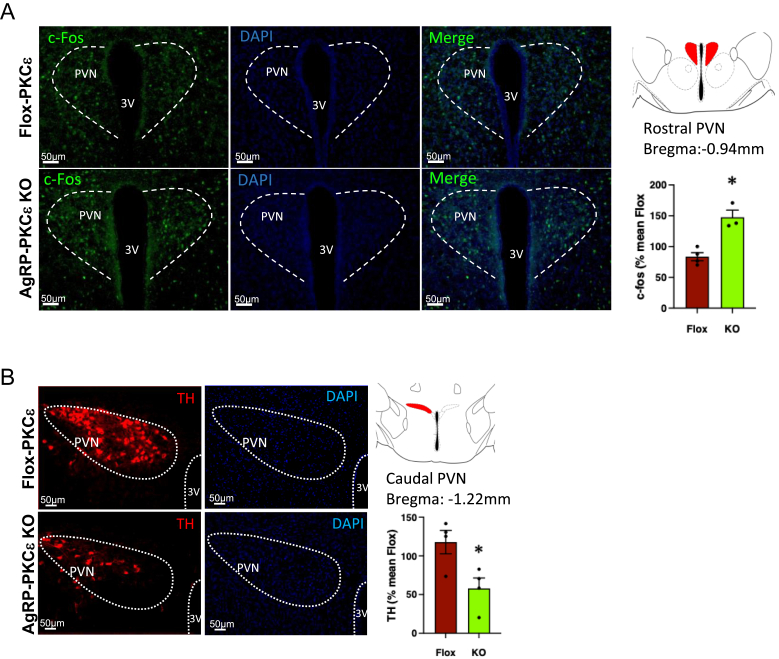
Effect of deletion of PKCe in AgRP neurons on c-FOS and tyrosine hydroxylase (Th) expression in the PVN. AgRP-PKCe KO mice and Flox-PKCe controls were challenged with glucose (1g/kg ip) and brains fixed after 30 min as for Fig. 3(**a**). Cryosections were subjected to immunofluorescence staining using antibodies against c-Fos (**a**) or tyrosine hydroxylase (TH) (**b**) and DAPI staining; cell nuclei were stained with DAPI (blue). 3V, 3^rd^ ventricle. Intensity of c-Fos and TH staining was quantified within the defined areas of interest in sections from 3 animals per group; Student’s t-test, ∗P< 0.05.

MARCKS is enriched both within axon terminals and dendritic spines, and its phosphorylation regulates actin-dependent processes required for cellular outgrowth [55]. While it does not appear to regulate presynaptic dynamics, its phosphorylation may play roles in synapse formation and dendritic arborization [55], which in turn could affect the way AgRP neurons modulate responses in other regions of the brain involved in glucose sensing. We next therefore interrogated the effects of glucose in further hypothalamic nuclei, examining c-Fos expression as a marker for neuronal activation. AgRP neurons have been shown to directly innervate the PVN from the ARC [56,57], and this connection has been implicated in both energy balance and acute stress responses [58]. PKCε deficiency in AgRP neurons resulted in greater c-Fos expression in in the PVN (Fig. 4A), whereas changes were less apparent in the DMH and no changes were observed in the VMH (Supplementary Fig. 3). We subsequently examined the effects of PKCε deletion in AgRP neurons on tyrosine hydroxylase (Th) expression in the PVN, as an indirect marker of sympathoexcitation [39,59] by immunostaining. A striking decrease was observed in Th expression (Fig. 4B). Taken together, our results implicate PKCε in the phosphorylation of MARCKS in AgRP neurons, which in turn alters the activation of neurons in the PVN in response to glucose and the subsequent sympathetic outflow, as indicated by diminished Th levels.

### 3.3 **PKC**ε deletion in AgRP neurons affects markers of sympathetic stimulation in adipose tissue

**Figure 5:**
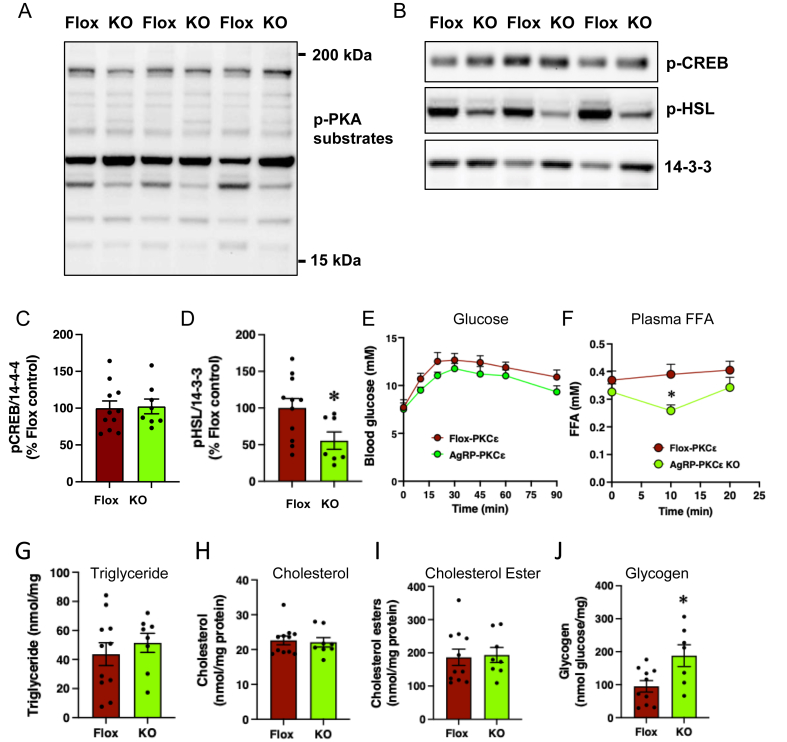
PKCe deletion in AgRP neurons reduces phosphorylation of HSL in WAT. (**a**) Phosphorylation of general PKA substrates, CREB and HSL in WAT in fat-fed Flox-PKCe and AgRP-PKCe KO (KO) mice determined by immunoblotting, 30 min after glucose challenge. (**b**) Phosphorylation of CREB and HSL, with densitometric analysis shown in (**c**) and (**d**); t=test, ∗P<0.05. Fat-fed mice were subjected to a pyruvate tolerance test, and blood glucose (**e**) and plasma FFAs (**f**) measured at the intervals shown. Levels of triglyceride (**g**), choleterol (**h**), choleterol esters (**i**) and glycogen were measured in livers from mice challenged as in (**a**); Student’s t-test, ∗P< 0.05.

Given the reduction in Th expression in the PVN, deletion of PKCε in AgRP neurons may affect peripheral tissues such as adipose tissue and liver via altered sympathetic stimulation [60], which could in turn affect HGP indirectly or directly. We first examined mRNA levels of markers of sympathetic activity in brown adipose tissue (BAT) from fat-fed mice. PKCε deletion in AgRP neurons resulted in mRNA levels trending lower for 6 out 7 marker genes in BAT compared to littermate controls (Supplementary Fig. 4a, ANOVA: Effect of genotype P < 0.001) although this was not accompanied by a change in UC1 protein level (Supplementary Fig. 4b) or BAT mass (Supplementary Fig. 4c). The expression of these genes was not diminished in gonadal white adipose tissue (WAT) while UCP1 protein and mass of this depot were also unaffected by PKCε deletion (Supplementary Fig. 4d-f). We next investigated changes in the phosphorylation of cAMP-dependent protein kinase (PKA) substrates as markers of acute sympathetic stimulation of WAT from glucose-challenged mice. Analysis of PKA substrate protein phosphorylation by immunoblotting indicated that some but not all PKA targets were less phosphorylated in adipose tissue from AgRP-PKCε KO mice (Fig. 5A). We examined this in more detail, observing that the phosphorylation of the transcription factor CREB was not affected (Fig. 5B,C), consistent with the lack of change in expression of the downstream marker genes (Supplementary Fig. 4d). However, deletion of PKCε in AgRP neurons did reduce the levels of phosphorylated hormone-sensitive lipase (HSL) in WAT from glucose-injected mice (Fig. 5B,D), suggesting a reduced rate of lipolysis. To investigate this further, we measured plasma FFA levels during a pyruvate tolerance test, which more specifically probes hepatic glucose output but also results in transient hyperglycaemia (Fig. 5E). Fat-fed AgRP-PKCε KO mice exhibited a lowering of plasma FFA which was not observed in littermate controls (Fig. 5F). Storage of lipids in the liver was not affected, in that hepatic triglyceride, cholesterol and cholesterol ester levels were unchanged (Figure 5G–I). however, hepatic glycogen levels were elevated in AgRP-PKCε KO mice (Fig 5J). Taken together, these findings are consistent with a reduction in acute sympathetic stimulation of WAT caused by PKCε deletion in AgRP neurons, resulting in decreased phosphorylation of HSL and hence release of FFAs, which in turn reduces hepatic glucose output.

### 3.4 Effects of PKCε deletion in AgRP neurons on IL-6 signalling in liver

**Figure 6:**
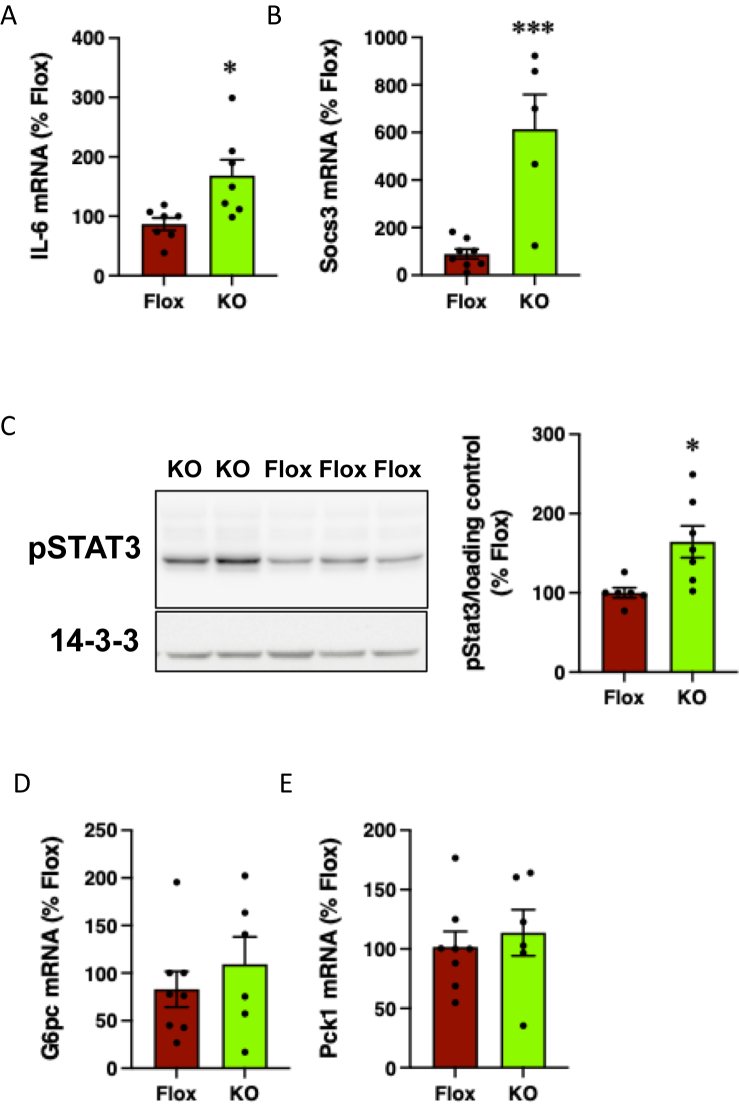
PKCe deletion in AgRP neurons increases IL-6 signalling in liver. AgRP-PKCe KO mice and Flox-PKCe controls were fasted overnight, challenged with glucose (1g/kg ip) and livers harvested after 4 h. Hepatic expression of (**a**) IL-6, (**b**) Socs3, (**d**) G6pc and (**e**) Pck1 mRNAs were determined by qRT-PCR. (**c**) Stat3 phosphorylation was determined by immunoblotting (left panel), quantified by densitometry (right panel) and corrected using a loading control (14-3-3 or total Stat3).

In addition to the modulation of sympathetic activity, the PVN mediates effects of the hypothalamus on the parasympathetic nervous system, including vagal tone [61]. Hyperpolarization of AgRP neurons by insulin leads to a reduction in vagally-mediated suppression of IL-6 release by Kupffer cells and activation of the IL-6/STAT3 pathway in hepatocytes, resulting in the decreased expression of gluconeogenic genes [8,9]. While this pathway has been described in the context of insulin action in the ARC, alterations in PKCε action in AgRP-PKCε KO mice may affect AgRP neuron function and thus glucose homeostasis in a glucose-dependent manner, independently of insulin. To test this, we examined IL-6 signalling in livers from mice injected with a glucose bolus. mRNA levels of IL-6 and Suppressor of Cytokine Signaling 3 (SOCS3), which is induced by IL-6 signalling, were increased in livers from AgRP-PKCε KO mice (Fig. 6A,B). The level of STAT-3 phosphorylation observed in glucose-challenged mice was also enhanced by PKCε deletion in AgRP neurons (Fig. 6C), suggesting greater activation of the hepatic IL-6/STAT-3 pathway in AgRP-PKCε KO mice. Intriguingly, however, this was not accompanied by a reduction in the mRNA levels of the gluconeogenic genes glucose-6-phosphatase (G6pc) and phosphoenolpyruvate carboxykinase (Pck1) (Fig. 6D,E), in contrast to the reported pSTAT3-dependent depletion of these mRNAs [8,9]. Together, these data suggest that the improved glucose tolerance observed in AgRP-PKCε KO mice may, at least partly, be explained by increased hepatic IL-6/STAT-3 signalling, although independently of changes in gluconeogenic gene expression.

### 3.5 Proteomic analysis of liver and adipose tissue from AgRP-PKCε KO mice

**Figure 7:**
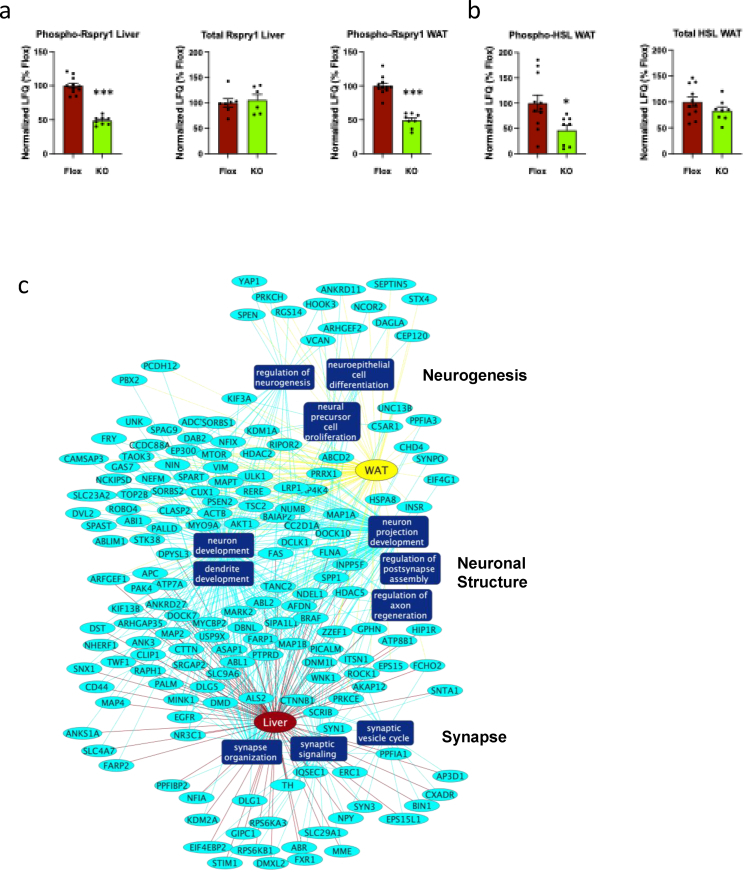

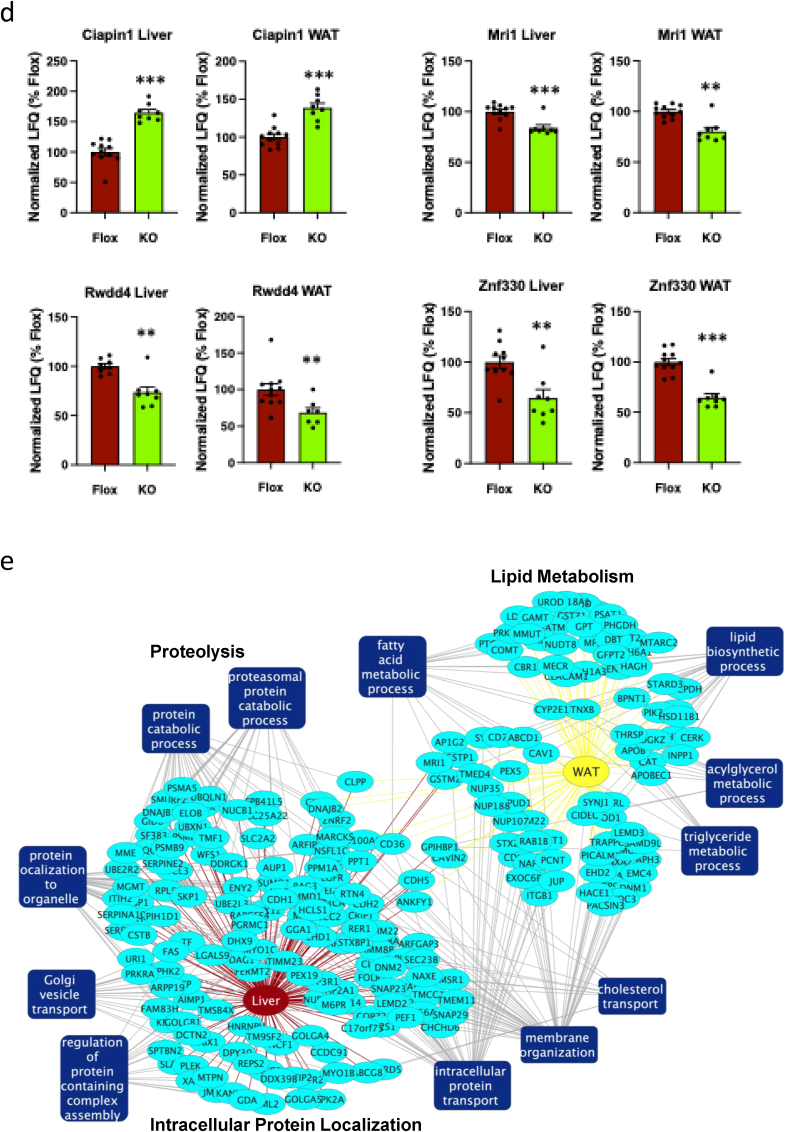
PKCe deletion in AgRP neurons has coincident effects in liver and WAT on both the phospho-proteome and proteome. Mice were challenged with glucose (1g/kg ip), tissues harvested after 30 min and liver and WAT subjected to proteomic and phospho-proteomic analysis. (**a**) Phosphorylation of Rspry1 at Ser50 and total protein in liver and WAT; ∗∗∗P<0.001 (**b**) phosphorylation of HSL Ser559 in WAT; ∗P<0.05. (**c**) Network generated using Cytoscape, indicating the enrichment of differentially phosphorylated proteins (ellipses) in liver and WAT according to Biological Process Gene Ontology terms related to neurons (rectangles). (**d**) Abundance of proteins similarly affected by PKCe deletion in liver and WAT. (**e**) Network indicating the enrichment of proteins in liver and WAT according to Biological Process Gene Ontology terms terms related to lipid metabolism, proteolysis and protein localization (rectangles).

To further examine the dynamic effects of PKCε deletion in AgRP neurons on liver and WAT, we used LC-MS/MS to analyse the phosphoproteomes of these tissues from fat-fed mice upon glucose challenge. We identified 13,004 and 13,494 unique phosphosites in liver and WAT respectively (Supplementary Tables 1 and 2). Strikingly, we identified Rspry1 Ser50 as the phosphosite most significantly affected by PKCε deletion in AgRP neurons in both liver and WAT, being downregulated by approximately 50% in each case (Fig. 7A, Supplementary Tables 1 and 2). This was not accounted for by changes in the total abundance of Rspry1, at least in liver (Fig. 7A, Supplementary table 3), although total Rspry1 protein could not be quantified in WAT (Supplementary table 4). Analysis of the Rspry1 Ser50 flanking sequence implicated reduced phosphorylation by the G protein-coupled receptor kinase (GRK) family, or by the calcium/calmodulin-dependent protein kinase 2 (Camk2) family, with 6 GRK members as well as Camk2A and 2 B among the top 15 predicted kinases, all within the 95th percentile (Supplementary table 5). These kinases are stimulated by adrenergic receptor activation, in agreement with sympathetic downregulation in AgRP-PKCε KO mice. In further support of a sympathetic mechanism, phosphorylation of the HSL (Lipe) peptide containing the phosphosite downregulated in WAT according to immunoblotting (Fig. 5E,G) was also found to be reduced by LC-MS/MS (Fig. 7B, Supplementary table 2). Phosphorylation of Creb1 Ser 133 in WAT and STAT3 Tyr 705 in liver were not detected. Finally, co-analysis of the phosphoproteins identified from liver and WAT (Supplementary Tables 1 and 2, genotype effect, P < 0.05) indicated proteins enriched for association with neuronal processes such as neurogenesis and synapse structure and function (Fig. 7C).

We also analysed the effects of PKCε deletion on the total proteome, quantifying 5763 and 5848 proteins in liver and WAT respectively from fat-fed AgRP-PKCε KO and littermate control mice (Supplementary Tables 4 and 5). When proteins were ranked by P value for the effect of genotype, 4 proteins in the top 15 from liver and WAT were found to be similarly regulated in both tissues: Ciapin1 was upregulated while Mri1, Rwdd4 and Znf330 were all downregulated in both liver and WAT from fat-fed AgRP-PKCε KO mice (Fig. 7D). On the one hand, this lends further support to changes in a common cell type, such as neurons participating in a circuit from upstream AgRP neurons, in both tissues. On the other hand, overrepresentation analysis indicated that proteins affected in liver were enriched for association with protein trafficking, localization and catabolism, whereas proteins affected in WAT were associated with fatty acid and glycerolipid metabolism (Fig. 7E). Altogether, PKCε deficiency in AgRP neurons is associated with proteomic and phosphoproteomic changes in liver and WAT supportive of PKCε-sensitive circuits linking the hypothalamus with these metabolic tissues.

## 4 Discussion

While several studies have confirmed a major role for PKCε in the modulation of glucose homeostasis [[25], [26], [27]], the sites and mechanisms of action involved remain controversial [30,31]. Here we examined the effects of PKCε deletion in AgRP neurons in the hypothalamic arcuate nucleus, demonstrating an improvement in glucose tolerance in longer term fat-fed mice, which was not explained by alterations in insulin secretion or insulin action. We also observed differences in the activation of specific regions of the hypothalamus in response to glucose, as well as in protein phosphorylation and metabolite levels in peripheral tissues. Our data are consistent with an effect of PKCε on central glucose-sensitive circuits that impact on peripheral glucose metabolism, by acutely altering the supply of lipolytic products from adipose tissue and thus modifying hepatic glucose output.

These findings extend our earlier studies of global and tissue-specific PKCε KO mice that highlighted distinct phenotypes related to glucose homeostasis, depending on the sites of deletion and the duration of fat diet feeding. We previously showed that whole body PKCε deletion results in improved glucose tolerance and insulin sensitivity [24,26,27] which were apparent after one week of high fat diet feeding, while beneficial effects on glucose-stimulated insulin secretion were only observed after six weeks, when β-cell dysfunction in fat-fed mice becomes more pronounced [26,27,62]. Deletion specifically in adipose tissue also resulted in improved glucose tolerance at one week, but this was not associated with greater insulin sensitivity compared to control mice, while deletion in liver did not result in improvement in glucose tolerance or insulin sensitivity at any time [27]. The site at which genetic PKCε deletion protected mice against early diet-induced insulin resistance was therefore not identified, and here we now examined the effects of PKCε deficiency in AgRP neurons, which have been implicated in centrally-mediated regulation of glucose metabolism. However, the glucose tolerance of AgRP-PKCε KO mice fed a high fat diet for one week was not different to that of littermate controls, suggesting that ablation of PKCε activity in AgRP neurons does not account for the improvement in insulin sensitivity observed in global PKCε KO mice in the early stages of a high fat diet. These data also indicate that any PKCε-mediated dysregulation of glucose homeostasis mediated in AgRP neurons does not occur in the early stage of fat-feeding.

However, we did observe partial protection against diet-induced glucose intolerance in AgRP-PKCε KO mice fed a high fat diet for eight weeks. In contrast to our findings made using global KO mice [24,26,27,62], this was not associated with an enhanced insulin response during the ipGTT. Therefore, although hypothalamic effects on β-cell function have been reported [7], PKCε action in AgRP neurons does not modulate insulin secretion in fat-fed mice, and another site of action must mediate the improved insulin response observed in global PKCε KO mice. In addition, our data from euglycaemic-hyperinsulinaemic clamps demonstrate that changes in insulin sensitivity do not account for the improved glucose tolerance of AgRP-PKCε KO mice. AgRP neurons are also glucose-sensitive, and our findings are therefore consistent with the modulation by PKCε of a glucose-dependent central effect on glucose metabolism, rather than of an insulin-dependent mechanism. In addition, while we did not observe any effect of PKCε deletion on body weight or composition, this is in agreement with global deletion of the kinase [24,26] and also with studies demonstrating that AgRP neuron function is not necessary for body weight maintenance under *ad libitum* feeding conditions [63].

While insulin stimulation causes hyperpolarization and silencing of AgRP neurons to reduce HGP acutely [8,64], a fall in glucose levels can promote depolarization in an AMPK-dependent manner [52,65]. In addition to promoting food intake and adaptations in energy expenditure, this may also enable restoration of glucose levels more directly by affecting glucose metabolism in peripheral tissues [6]. While increased activation of PKCε in AgRP neurons of fat-fed mice could conceivably enhance neuron activation and thus inappropriately stimulate HGP at higher glucose concentrations, our data from GT1-7 cells suggests that PKCε does not modulate the effects of glucose on the activation state of AMPK. While other intracellular targets causing disruption of glucose-mediated inhibition are possible, our findings in cultured cells and *in vivo* are instead consistent with an effect of PKCε deletion on MARCKS phosphorylation. Changes in the phosphorylation of this protein control filamentous actin cross-linking at the plasma membrane and affect several aspects of nerve cell function [55]. This may account at least in part for the increased activation specifically of neurons in the PVN, implicated in glucose sensing [6], in AgRP-PKCε KO mice upon glucose challenge.

The reduction in expression of Th observed in the PVN of AgRP-PKCε KO mice may contribute to the enhanced glucose tolerance observed in these animals, by resulting in a lower sympathetic outflow to peripheral tissues. Because PKCε deletion was unconditional, we cannot exclude the possibility of a developmental effect that causes congenital Th downregulation in the PVN. Further studies will be required to clarify this. Nevertheless, reduced sympathetic stimulation of BAT would inhibit the expression of thermogenic genes [39,66,67], and the reduced mRNA levels we observed for several marker genes in BAT from AgRP-PKCε KO mice are thus in alignment with the lower Th levels, consistent with the control of BAT by neurons in the ARC via the PVN [39]. However, these alterations in gene expression did not translate into changes in UCP1 protein expression, BAT mass or total body weight, suggesting any change in thermogenic potential and energy expenditure was latent. It is possible that the modification in gene expression requires a longer time to manifest in phenotypic changes, or that compensatory mechanisms elsewhere in the body counteract these effects. Further investigation under additional stressors, such as cold exposure or altered nutrient intake, may be required to reveal these latent effects.

Meanwhile, the improved glucose tolerance we observed is more likely to be related to reduced sympathetic outflow to WAT. We did not observe changes in thermogenic gene expression in gonadal WAT from AgRP-PKCε KO mice, or changes in the upstream transcription factor CREB, which is in keeping with the relatively low induction of thermogenic genes in this depot [68]. In contrast, however, the phosphorylation of HSL was diminished in gonadal WAT of glucose-challenged AgRP-PKCε KO mice, suggesting that PKCε-modulated glucose sensing in the hypothalamus results in the acute modulation of lipolysis in WAT. In agreement, we saw evidence of a reduction in the release of FFAs from WAT by lipolysis, which is known to promote HGP [[69], [70], [71]] and which may contribute to an increase in HGP in fat-fed mice upon impaired central glucose sensing and increased sympathetic stimulation. The higher glycogen content in AgRP-PKCε KO mice is also compatible with reduced glucose output. Our data are therefore consistent with a reduction in HGP by deletion of PKCε in AgRP neurons and the restoration of glucose-mediated events in the hypothalamus, resulting in a reduction in sympathetic outflow from the PVN and in diminished lipolysis. This could occur in the absence of an improvement in insulin sensitivity, in keeping with our clamp data. Our previous studies of PKCε KO mice also support an indirect effect of PKCε on lipolysis in WAT. Firstly, whole body PKCε KO mice exhibited reduced plasma FFA levels upon extended fasting, and did not lose as much WAT mass as wildtype mice under these conditions [72]. However, deletion of the kinase specifically in adipose tissue did not result in any change in isoproterenol-stimulated fatty acid release from primary adipocytes, nor in its inhibition by insulin [27]. These studies, taken together, are consistent with a central role for PKCε in the regulation of lipolysis in a glucose-dependent manner. However, a limitation of the current work is the absence of a direct demonstration of altered sympathetic nerve activity, and alternative mechanisms such as endocrine regulation, indirect autonomic system action or developmental alterations may play a role. Another limitation concerns the downregulation of MARCKS phosphorylation in the hypothalamus, which was not demonstrated to be specific to AgRP neurons, and could be a more indirect effect of PKCε deletion.

Other neuronal pathways, such as parasympathetic regulation may also contribute to such an effect, since enhanced parasympathetic activity increases glucose tolerance by decreasing HGP [73]. Thus, an alternative circuit linking AgRP neurons to the suppression of HGP, potentially via the PVN [61], involves reduced vagal stimulation of Kupffer cells in the liver, resulting in increased IL-6 release and stimulation of STAT3 signalling in hepatocytes to downregulate expression of the gluconeogenic enzyme G6pc [9]. Hepatic expression of IL-6 and SOCS3 and phosphorylation of STAT3 were augmented in glucose-challenged mice, in agreement with a role for this pathway in glucose-mediated central regulation of HGP. However, in this case the endpoint is unlikely to be the suppression of gluconeogenic enzyme levels, because the expression of Pck1 and G6pc was not changed. Further investigation is required to determine to what extent this pathway plays a direct role in the improved glucose tolerance of AgRP-PKCε KO mice, and to elucidate the responsible downstream effects of hepatic IL-6/STAT3 signalling.

Investigation of the total proteomes in liver and WAT from AgRP-PKCε KO and control mice highlighted several proteins that were differentially regulated by genotype in a similar way in both tissues. While these proteins (Ciapin1, Mri1, Rwdd4 and Znf330) have not been associated with metabolic function, two of the four (Rwdd4 and Znf330) have been associated with neuropathology [74]. This is consistent with the possibility that such proteins originate in neuronal tissue and that the effects of PKCε deletion in AgRP neurons on liver and WAT are in part mediated by sympathetic circuits. In further support, our phosphoproteomic studies remarkably identified the same phosphoprotein, Rspry1, as the most significantly affected by genotype in both tissues. While its function is unknown, Rspry1 Ser 50 phosphorylation has been detected in mouse synaptic membranes [75] and the phosphosite appears to be a target for adrenergically-stimulated kinases. A neuronally-mediated mechanism was also reinforced by overrepresentation analysis of PKCε-sensitive phosphoproteins which highlighted several biological processes linked to neuron function. Changes in the total proteome of WAT from AgRP-PKCε KO mice were associated with lipid metabolism, consistent with modulation of fatty acid fluxes. However, the changes related to proteostasis that we observed in the liver are more difficult to interpret metabolically, but may lie downstream of alterations either in sympathetic or parasympathetic input and indirectly promote glucose homeostasis.

In summary, we have shown that PKCε deletion specifically in AgRP neurons of fat-fed mice improves glucose tolerance, but unexpectedly this does not result from changes in insulin secretion or insulin sensitivity. Instead, we present evidence that PKCε modulates glucose sensing in the hypothalamus, which may involve alterations in MARCKS phosphorylation in AgRP neurons and thus changes in the circuits downstream of the ARC leading to neuronal stimulation of WAT and liver. While any direct effects on the liver do not involve changes in expression of gluconeogenic genes and remain to be clarified, we hypothesize that a reduction in lipolysis in WAT downstream of PKCε deletion in the brain contributes to a decrease in HGP. The relative contributions of these mechanisms also remain to be determined.

## CRediT authorship contribution statement

**Amanda E. Brandon:** Methodology, Investigation, Formal analysis. **Chenxu Yan:** Visualization, Investigation. **Xuan Zhang:** Investigation. **Chi Kin Ip:** Writing – review & editing, Visualization, Investigation. **Zhongmin Gao:** Investigation. **Nicola J. Lee:** Writing – review & editing, Investigation, Formal analysis. **Oana C. Marian:** Writing – review & editing, Investigation, Formal analysis. **Alex Perez:** Investigation, Formal analysis. **Anthony S. Don:** Resources, Methodology. **Herbert Herzog:** Resources, Methodology. **Lewin Small:** Writing – review & editing, Investigation, Formal analysis, Data curation. **Yan-Chuan Shi:** Writing – review & editing, Supervision, Resources, Methodology, Funding acquisition. **Carsten Schmitz-Peiffer:** Writing – original draft, Visualization, Supervision, Resources, Project administration, Investigation, Funding acquisition, Formal analysis, Data curation, Conceptualization.

## Funding

This research was supported by the National Health and Medical Research Council (NH&MRC) APP1081869, APP2019722 to CSP, APP1162276, APP2019361 to YCS, Perpetual Impact Philanthropy Program, Hillcrest Foundation, IPAP 2019/0252, 2020/0067, 2021/0140, 2022/0041, 2023/0163) to CSP, and a Diabetes Australia DART grant (Y20G-Shiy) to YCS.

## Declaration of competing interest

The authors declare that they have no known competing financial interests or personal relationships that could have appeared to influence the work reported in this paper.

## Data Availability

Data will be made available on request.
