# Supplementary material for: Protein kinase C epsilon deletion in AgRP neurons modulates hypothalamic glucose sensing and improves glucose tolerance in mice
Source: Mol Metab. 2026 Jan 13;104:102320. doi: 10.1016/j.molmet.2026.102320 (PMC12860745; doi:10.1016/j.molmet.2026.102320)
Supplement: Multimedia component 2 [file mmc2.pptx]

## Slide 1
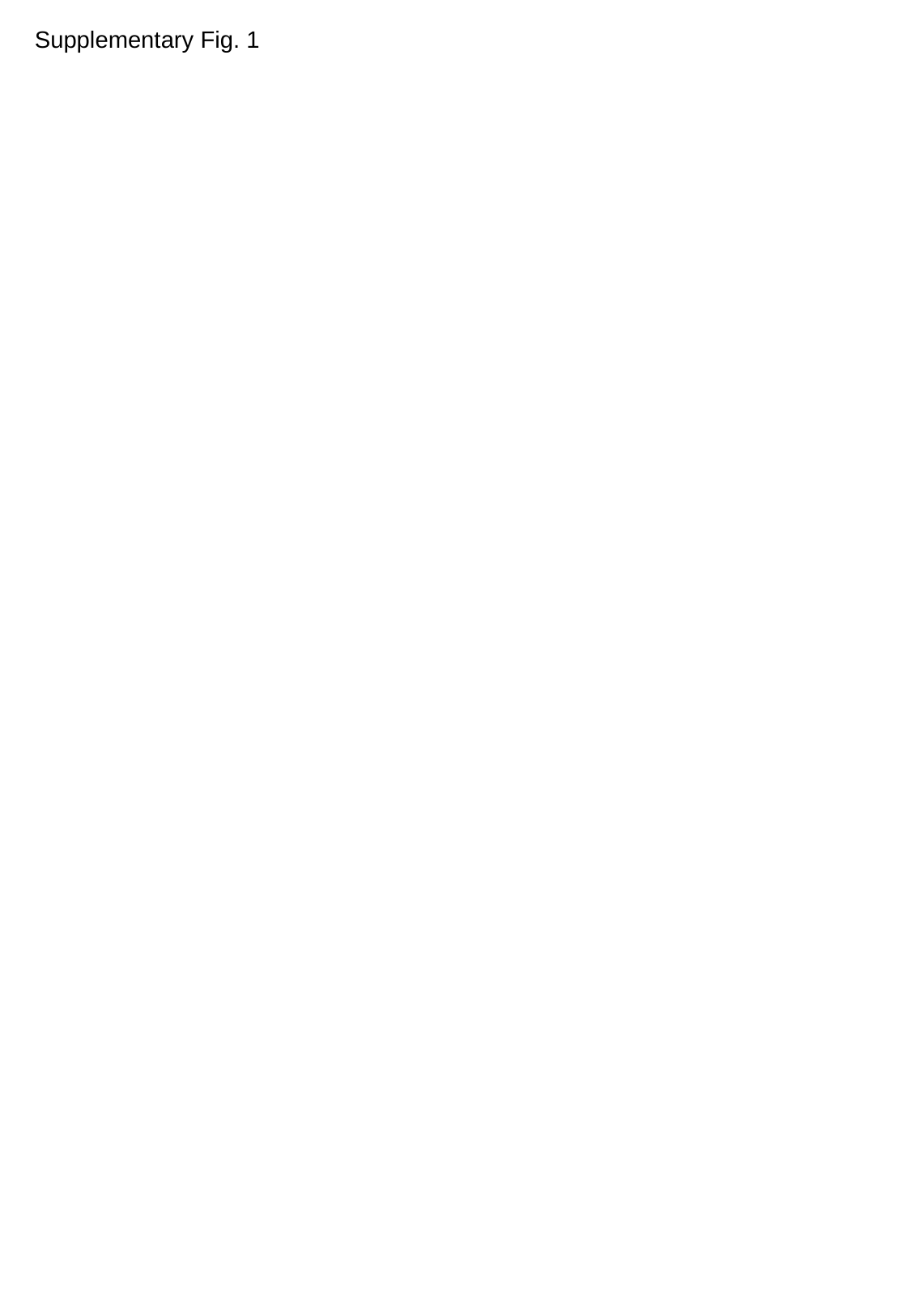

Supplementary Fig. 1

## Slide 2
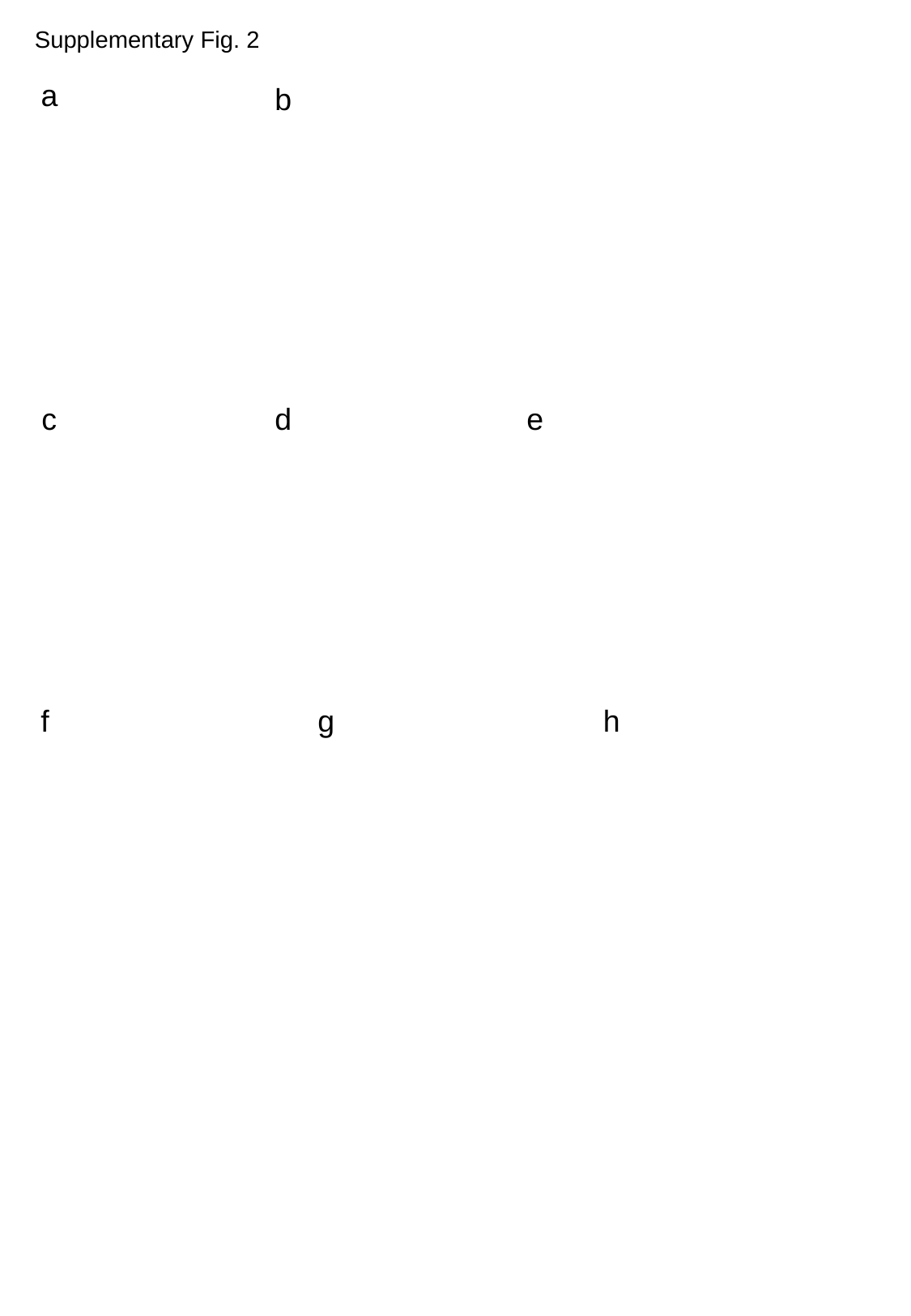

Supplementary Fig. 2
a
b
c
d
e
f
g
h

## Slide 3
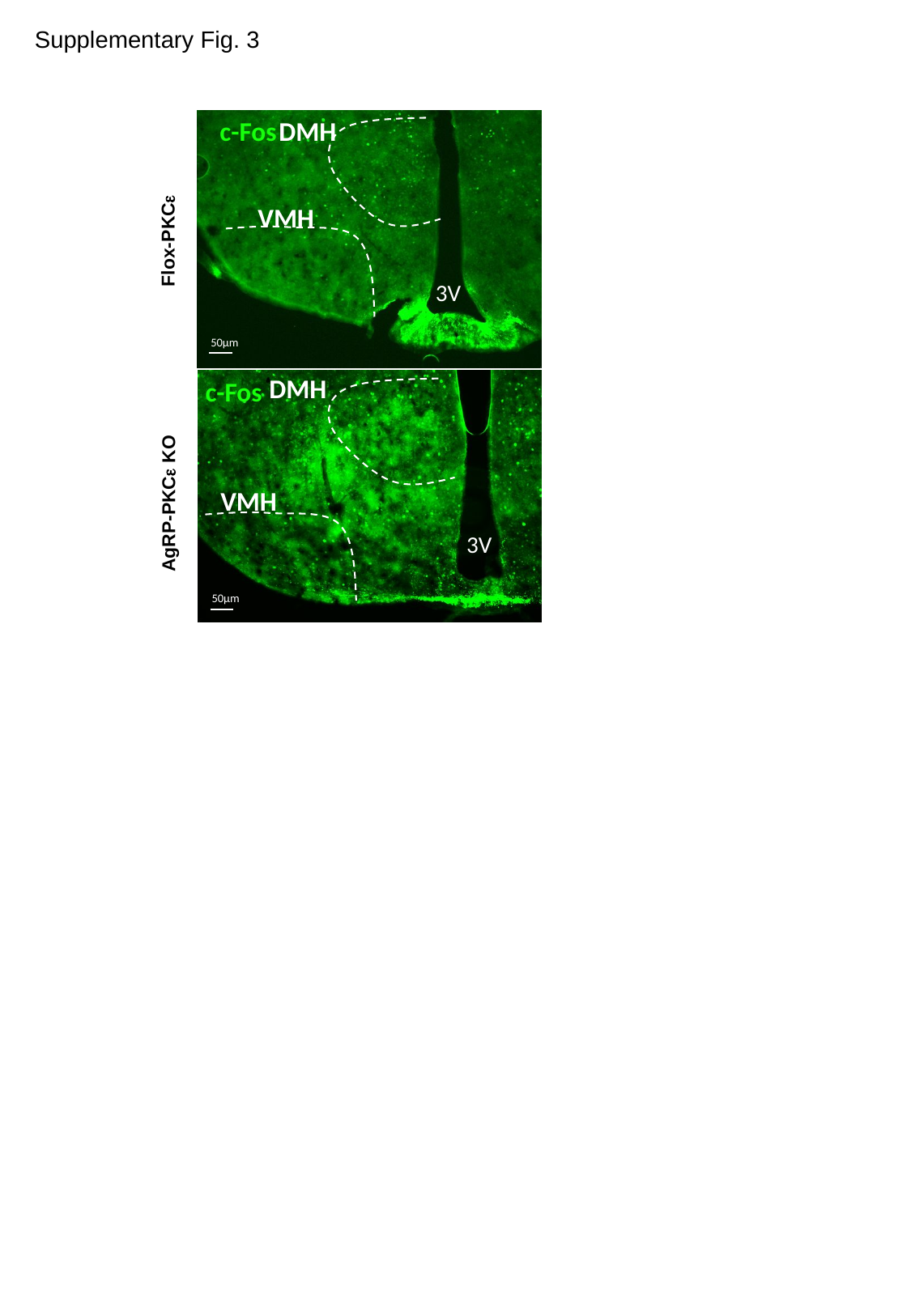

Supplementary Fig. 3
DMH
c-Fos
VMH
Flox-PKCe
3V
50μm
DMH
c-Fos
VMH
AgRP-PKCε KO
3V
50μm

## Slide 4
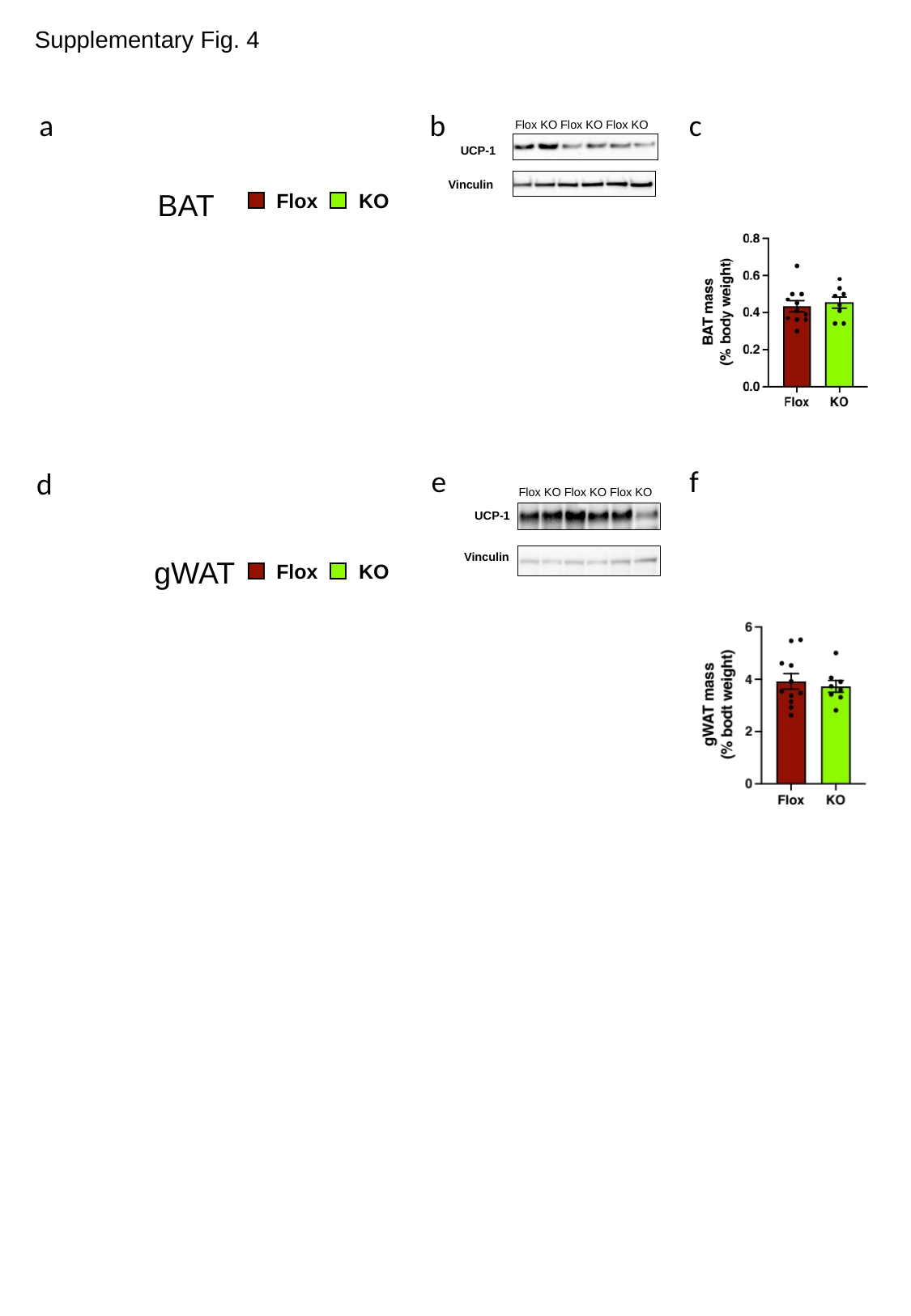

Supplementary Fig. 4
a
b
c
Flox KO Flox KO Flox KO
UCP-1
Vinculin
BAT
Flox
KO
e
f
d
Flox KO Flox KO Flox KO
UCP-1
Vinculin
gWAT
Flox
KO
